# Using television shows to teach communication skills in internal medicine residency

**DOI:** 10.1186/1472-6920-9-9

**Published:** 2009-02-03

**Authors:** Roger Y Wong, Sadra S Saber, Irene Ma, J Mark Roberts

**Affiliations:** 1 Postgraduate Medical Education, Department of Medicine, University of British Columbia, Vancouver, Canada

## Abstract

**Background:**

To address evidence-based effective communication skills in the formal academic half day curriculum of our core internal medicine residency program, we designed and delivered an interactive session using excerpts taken from medically-themed television shows.

**Methods:**

We selected two excerpts from the television show *House*, and one from *Gray's Anatomy *and featured them in conjunction with a brief didactic presentation of the Kalamazoo consensus statement on doctor-patient communication. To assess the efficacy of this approach a set of standardized questions were given to our residents once at the beginning and once at the completion of the session.

**Results:**

Our residents indicated that their understanding of an evidence-based model of effective communication such as the Kalamazoo model, and their comfort levels in applying such model in clinical practice increased significantly. Furthermore, residents' understanding levels of the seven essential competencies listed in the Kalamazoo model also improved significantly. Finally, the residents reported that their comfort levels in three challenging clinical scenarios presented to them improved significantly.

**Conclusion:**

We used popular television shows to teach residents in our core internal medicine residency program about effective communication skills with a focus on the Kalamazoo's model. The results of the subjective assessment of this approach indicated that it was successful in accomplishing our objectives.

## Background

Effective communication skills are integral to patient care as they enhance patient satisfaction, adherence to treatment and health outcomes [1]. It is therefore inherent that teaching communication skills is an important component in undergraduate and postgraduate medical education. In fact, accreditation bodies such as the Accreditation Council for Graduate Medical Education (ACGME) in the United States [2] and the CanMEDS framework mandated by the Royal College of Physicians and Surgeons (RCPSC) in Canada [3] have declared communication as one of the core competencies and roles of physicians. Several guidelines [4-6], models [7,8], and consensus statements [9-11] have been developed to guide medical educators, learners and physicians in developing sound communication skills. However, challenges still remain in addressing patient-physician communication in medical education. These include inadequate attention and time given to educating undergraduate and postgraduate learners on communication skills, difficulties in implementing standardized programs of communication skills in formal medical curricula, as well as lack of objective evaluation of the efficacy of such programs [12-16]. These challenges, in turn, have resulted in deficiencies in patient-physician communication in medical practice. Discussing end-of-life issues, delivering bad news, attending to psychosocial aspects of patients, and disclosing errors or adverse events are some examples of areas where pitfalls in physician communication have been documented [17-19].

Recognizing many of those issues, in 1999 representatives from major medical educational and professional organizations gathered in a conference in Kalamazoo, Michigan, with the objective of outlining a set of universal guidelines by which effective communication skills can be taught, evaluated, and utilized in medical education and practice [9]. By the end, the collaboration of the experts from a variety of backgrounds and disciplines in medical education and practice produced a comprehensive and evidence-based model which outlines seven essential elements in doctor-patient communication, and is now widely used in medical education. The seven elements listed in the model, in brief, are: building a relationship, opening the discussion, gathering information, understanding the patient's perspective, sharing information, reaching agreement on problems and plans, and finally, providing closure [9].

So far, no formal structured teaching methods of the Kalamazoo model exist in the literature. We intended to reinforce the importance of effective communication with particular attention to the Kalamazoo model by means of cinemeducation. This innovative approach was first reported in 1994 and involves the use of clips from popular films and television shows in education [20]. Since then, this approach has been successfully used in different areas of medical education including psychiatry [21-23], family medicine [24], internal medicine residency programs [25] as well as in undergraduate medical [26,27] and nursing [28] education.

Our objective was to assess the efficacy of cinemeducation (television shows in our case) for teaching communication skills using the Kalamazoo model, which has not been included in previous studies. The primary endpoint was the self-rated understanding of the evidence-based Kalamazoo model on communication. The secondary endpoint was the self-rated comfort levels of residents in three challenging clinical scenarios. These were: addressing end-of life issues with terminally ill patients, attending to psychosocial aspects of illness, and disclosing errors and adverse events.

## Methods

### Setting

The core internal medicine residency training program at the University of British Columbia (UBC) is a 3-year program with 115 residents. These residents had not received formal instruction on the Kalamazoo model of communication. In addition to clinical rotations, formal curriculum delivery is done through academic half days (AHD) which are held weekly for four hours and aimed to cover the topics in various medical specialties as well as the non-medical expert competencies such as communication, collaboration, bioethics, evidence-based medicine, and quality improvement, etc. On average, about 70 residents attend any AHD whereas the remainder is excused due to post-call days, out of town rotations, vacations, etc. We received approval from the UBC residency training committee to develop and implement the communication curriculum/testing and publish our experience.

### The Communication Skills Seminar and Viewing Session

We organized a one-hour interactive session on communication during AHD teaching time that targeted residents in all three post-graduate years (PGY). Prior to initiating the AHD session, a baseline assessment (pre test) was completed by the residents, which contained questions on the various aspects of communication skills based on the essential elements in the Kalamazoo consensus statement on communication. Then a brief didactic presentation was given on effective communication competencies. Subsequently, we featured two scenarios excerpted from the first season of the television show *House*, and one from the first season of *Gray's Anatomy*. The episodes were selected by one of the chief medical residents who screen played the entire first season of both shows. The nature of the episodes were carefully chosen to depict important and sensitive situations with respect to doctor-patient communication. These excerpts represented 3 clinical scenarios that are articulated in the core competencies of effective communication as documented by the educational accreditation body, namely, addressing end-of life issues, attending to psychosocial aspects of illness, and disclosing medical errors. Each feature presentation was followed by 7 questions that mirrored the seven elements in the Kalamazoo's model (see Appendix). This was followed by an interactive reflection period where residents were encouraged to input their ideas on the excerpts verbally and discuss what they would have done differently in each of the featured scenarios. Finally a post-test was administered.

### Data Collection

The standardized questions were designed by the AHD director and peer-reviewed by faculty members and senior residents to establish face and content validity. The questions were specifically devised to address the end-points we previously defined (see Appendix).

The residents' responses throughout the academic session (with the exception of the reflection segment) were collected via Personal Response System (PRS) transmitters [29]. We explained to the residents that, by responding through the PRS, they have granted their verbal consent to submit their data for group analysis and reporting. All questions and potential responses were programmed into a central notebook computer prior to the session. Each resident was handed a PRS transmitter. A 10-seocnd interval per question was included for residents to key in their responses. All responses were based on a five-point Likert scale (1 = poor, 2 = fair, 3 = good, 4 = very good, 5 = excellent).

The collected data was then statistically analyzed by comparing the median values of all Likert responses (score range 0–5) before and after the AHD session using Wilcoxon paired signed ranked tests. Paired proportions were compared using McNemar's tests. These non-parametric tests were chosen because our data did not follow the normal distribution. All analyses were performed using SAS statistical software, version 9.1 (SAS Institute Inc, Cary, NC).

## Results

A total of 64 residents attended the communication session, and 43 (67%) responded using the PRS transmitters (21 PGY-1, 15 PGY-2, 7 PGY-3).

**Figure 1:**
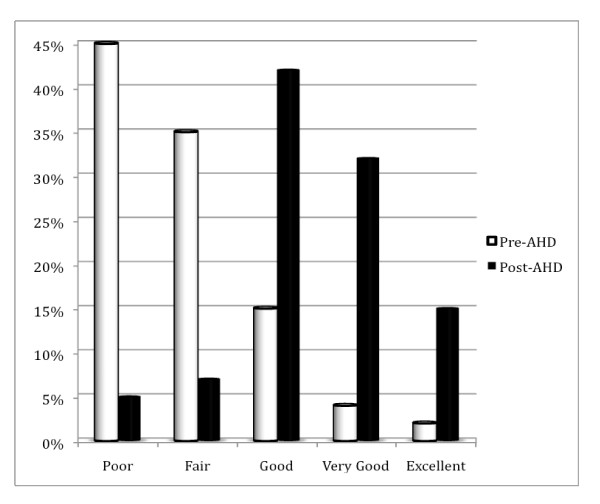
**Residents' self-rated understanding of an evidence-based model of effective communication before and after the academic half day (AHD) session**.

**Figure 2:**
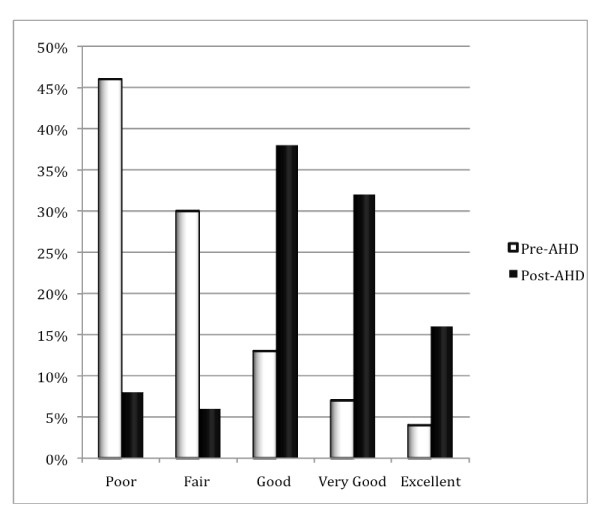
**Residents' self-rated comfort levels in applying evidence-based and effective communication skills to patient encounters before and after the academic half day (AHD) session**.

The residents who responded demonstrated significant improvements in their self-rated understanding of effective communication (Figure 1). At baseline (pre test), only 23% of respondents indicated that they had a good, very good or excellent understanding of an evidence-based communication model such as the Kalamazoo, whereas after the session, this proportion improved to 93% in the post test (P < 0.0001). Similarly, the residents' self-reported comfort in applying an evidence-based communication model to actual clinical encounters improved substantially (Figure 2), from 23% who indicated good, very good or excellent level in the pre test to 88% post test (P < 0.0001).

The AHD session also had positive effects in reinforcing to our residents the seven individual communication competencies listed in the Kalamazoo model. In fact, the residents' self-rated understanding of all the seven skills improved after the session, with statistical improvement in 6 out of 7 competencies in the Kalamazoo (Table 1). Resident understanding improved from a median of 3 (good) to 4 (very good) in every competency.

**Table 1:** Self-reported understanding of the essential elements in the Kalamazoo model of communication pre and post the academic half day (AHD) session.

**Competencies in the Kalamazoo Model**	**Pre-AHD: Median (IQR)**	**Post-AHD: Median (IQR)**	**P Value**
Build a relationship	3 (2–4)	4 (3–4)	0.005
Open the discussion	3 (2–4)	4 (3–4)	0.001
Gather information	3.5 (3–4)	4 (3–4)	0.13
Understand the patient's perspective	3 (3–4)	4 (3.5–4)	<0.001
Share information	3 (3–4)	4 (4–4)	<0.001
Reach agreement on problems and plans	3 (3–4)	4 (3–4)	0.005
Provide closure	3 (2–4)	4 (3–4)	<0.001

IQR = interquartile range.

Furthermore, the comfort levels of our residents in all three challenging clinical scenarios also increased significantly after the AHD session (Table 2). Specifically, comfort levels in addressing end-of-life issues and disclosure of adverse events/errors increased from a median of 2 (fair) to 3 (good).

**Table 2:** Self-reported comfort levels in 3 clinical scenarios pre and post the academic half day (AHD) session.

**Clinical Scenarios**	**Pre-AHD** **Median (IQR)**	**Post-AHD** **Median (IQR)**	**P Value**
Address end-of-life issues	2 (1–3)	3 (3–4)	<0.001
Attention to psychosocial aspects of illness	3 (2–3)	3 (3–4)	0.001
Disclosure of error or adverse event	2 (2–3)	3 (2–4)	0.001

IQR = interquartile range.

Finally resident satisfaction at the completion of the session was encouraging as 80% indicated that they would like to participate in this form of communication teaching in the future.

## Discussion

We developed and implemented a focused teaching session on evidence-based and effective communication skills as part of the formal academic half day curriculum using clips from popular television shows. This was done in conjunction with a brief discussion of the Kalamazoo consensus statement. We observed significant improvements in the residents' understanding of the essential elements of effective communication and their comfort levels in selected clinical scenarios that were deemed challenging.

Cinemeducation is an effective tool that can enhance teaching as it provides a dynamic and humanistic depiction of clinical situations to audiences, captures their attention, and engages them in the emotional experience [20]. This tool has become increasingly popular in medical education with the majority of its use in teaching about psychosocial aspects of illnesses and specific symptom presentations as well as other areas such as professionalism and therapeutic managements. Doctor-patient communication in medicine is also a key area that involves a great deal of art, emotion, and humanistic facets. While others have addressed interview skills and communication through cinemeducation in more specific clinical contexts [25,26,30,31] our session focused on generic communication competencies. The selected clips well complemented the didactic portion of the seminar by depicting clinical scenarios where the communication skills of doctors and residents were at play. In other words, each scenario featured an encounter where patient-physician communication was deficient in one or more competencies of the Kalamazoo model. This made it possible to realize the importance of effective communication, and enabled the residents to visualize realistic situations where the Kalamazoo model's competencies that were introduced in the preceding section could be applied. This novel approach proved to be a success as it provided for both an effective and an enjoyable experience, and helped in accomplishing our objectives of reinforcing the understanding and application of an evidence-based model of sound communication skills, according to the residents' feedback.

We recognize our educational intervention was brief (a one-hour session including presentation of the Kalamazoo model and some video excerpts). The long term impact of our intervention is unclear. Future studies are needed to compare cinemeducation with other educational approaches (such as role-playing and/or standardized patient encounters) in improving communication skills.

While this project had promising results, there are some limitations in the study that should be noted. This study is not a randomized controlled trial, lacked a control group, and is based on subjective assessments of respondents. Future work should consider more objective assessments. Also, since our AHD session involved a combination of introducing the Kalamazoo model didactically and cinemeducation, it is difficult for us to definitively attribute impact to the individual components of the AHD session that might have contributed to the pre-post differences observed. Furthermore, the study was done in one site with a relatively small sample size, and the response rates were less than ideal. In fact, the post test data was collected immediately after the intervention, making the observed gains potentially more optimistic. There could also be potential baseline differences between responders and no responders. While our residents indicated that this approach was useful in reinforcing effective communication skills, the study assessment was only done in an educational setting, and its efficacy with regards to real clinical situations remains unknown. The expansion of the study in the future to include more residents from upcoming years, and possibly the involvement of other programs or sites could help improve the generalizability of our findings. In addition, a desirable future step would be to take our study further into broader and more practical contexts such as clinical rotations in the internal medicine curriculum as well as clinical practices of the program graduates. In other words, while the results of our subjective assessment by the residents was encouraging, developing ways to assess the efficacy of our approach in real clinical encounters objectively, for instance by OSCE-style assessments, would further validate our findings. Finally, it might be interesting to collect qualitative data during the open-ended reflection period whereby residents interactively discussed the communication scenarios.

## Conclusion

To address effective communication skills and present the Kalamazoo's evidence-based model of doctor-patient communication in our academic half day curriculum, we designed a seminar which utilized three excerpts from the popular television shows *House *and *Gray's Anatomy*. The comparison of the survey results taken from the residents before and after the session showed that their understanding in the Kalamazoo model improved significantly, as did their comfort in all three selected challenging clinical scenarios.

## Competing interests

The authors declare that they have no competing interests.

## Authors' contributions

RYW was the principal investigator of the study, developed and delivered the seminar, and supervised the data collection, analysis, and drafting of the paper. SSS was responsible for literature review and drafting the paper. IM was responsible for data analysis and producing the results. Both IM and JMR also contributed by reviewing and improving the paper. All authors read and approved the final manuscript.

## Appendix

Standardized questions used during academic half day (AHD) session:

Pre AHD assessment:

Please rate your comfort level on:

1. Understanding of an evidence-based model of sound communication skills.

2. Application of an evidence-based model of sound communication skills to actual patient encounters.

Please rate your understanding of the skills to:

3. Build a relationship during interview.

4. Open the discussion.

5. Gather information.

6. Understand the patient's perspective.

7. Share information.

8. Reach agreement on problems and plans.

9. Provide Closure.

Please rate your comfort level on:

10. Addressing end-of-life issues.

11. Attention to psychosocial aspects of illness.

12. Disclosure of error or adverse event.

Assessment after showing each television clip:

How well did the doctor(s) do to:

1. Build a relationship.

2. Open the discussion.

3. Gather information.

4. Understand the patient's perspective.

5. Share information.

6. Reach agreement.

7. Provide closure.

Post AHD assessment:

Please rate your comfort level on:

1. Understanding of an evidence-based model of sound communication skills.

2. Application of an evidence-based model of sound communication skills to actual patient encounters.

Please rate your understanding of the skills to:

3. Build a relationship during interview.

4. Open the discussion.

5. Gather information.

6. Understand the patient's perspective.

7. Share information.

8. Reach agreement on problems and plans.

9. Provide Closure.

Please rate your comfort level on:

10. Addressing end-of-life issues.

11. Attention to psychosocial aspects of illness.

12. Disclosure of error or adverse event.

Please rate your level of agreement:

13. I would like to participate in this kind of communication teaching in the future.

## Pre-publication history

The pre-publication history for this paper can be accessed here:


